# Role of Air Bubble Inclusion on Polyurethane Reaction Kinetics

**DOI:** 10.3390/ma15093135

**Published:** 2022-04-26

**Authors:** Cosimo Brondi, Mercedes Santiago-Calvo, Ernesto Di Maio, Miguel Ángel Rodríguez-Perez

**Affiliations:** 1 Dipartimento di Ingegneria Chimica, Dei Materiali e Della Produzione Industriale, University of Naples Federico II, P.le Tecchio 80, 80125 Naples, Italy; cosimo.brondi@unina.it; 2 Cellular Materials Laboratory (CellMat), Condensed Matter Physics Department, Faculty of Science, Campus Miguel Delibes, University of Valladolid, Paseo de Belén, 7, 47011 Valladolid, Spain; mercesc@fmc.uva.es (M.S.-C.); marrod@fmc.uva.es (M.Á.R.-P.); 3 foamlab, University of Naples Federico II, P.le Tecchio 80, 80125 Naples, Italy

**Keywords:** polyurethane foams, nucleation, aeration, gelling, blowing

## Abstract

In this study, we investigated the influence of mixing conditions on the foaming process of water blown polyurethane (PU) foams obtained at different mixing speeds (50, 500, 1000 and 2000 rpm). In particular, the morphological evolution during the foaming process, in terms of the bubble size and bubble density, was studied via optical observations, while the effects on the reaction kinetics were monitored using in situ FTIR spectroscopy. At the slow mixing speed (50 rpm), no air bubbles were included and the early foaming process was characterized by the formation of new bubbles (CO_2_ nucleation), provided by the blowing reaction. Later on, it was observed that the coalescence affected the overall foaming process, caused by the gelling reaction, which was inhibited by the indigent mixing conditions and could not withstand the bubbles expansion. As a result, a PU foam with a coarse cellular structure and an average bubble size of 173 µm was obtained. In this case, the bubbles degeneration rate, dN/dt, was −3095 bubble·cm^−3^·s^−1^. On the contrary, at 500 rpm, air bubbles were included into the PU reaction system (aeration) and no formation of new bubbles was observed during the foaming process. After this, the air bubbles underwent growth caused by diffusion of the CO_2_ provided by the blowing reaction. As the gelling reaction was not strongly depleted as in the case at 50 rpm, the coalescence less affected the bubble growth (dN/dt = −2654 bubble·cm^−3^·s^−1^), leading to a PU foam with an average bubble size of 94 µm. For the foams obtained at 1000 and 2000 rpm, the bubble degeneration was first affected by coalescence and then by Ostwald ripening, and a finer cellular structure was observed (with average bubble sizes of 62 µm and 63 µm for 1000 rpm and 2000 rpm, respectively). During the first foaming stage, the coalescence was less predominant in the bubble growth (with dN/dt values of −1838 bubble·cm^−3^·s^−1^ and −1601 bubble·cm^−3^·s^−1^, respectively) compared to 50 rpm and 500 rpm. This occurrence was ascribed to the more balanced process between the bubble expansion and the PU polymerization caused by the more suitable mixing conditions. During the late foaming stage, the Ostwald ripening was only responsible for the further bubble degeneration (with dN/dt values of −89 bubble·cm^−3^·s^−1^ and −69 bubble·cm^−3^·s^−1^, respectively).

## 1. Introduction

PUs are materials used in a wide range of industrial sectors, such as in appliances, furniture and the automotive and construction industries [1,2,3]. According to their specific chemistry, they can be commercially available as solid thermoplastics (TPU) or as foams [4]. PU foams can be further classified as flexible foams (used for applications such as mattresses and automotive seats) or rigid foams (used for applications such as thermal insulation and structural materials) [1,2,3,4]. PUs are typically prepared by *mixing* two reactants, namely a polyether polyol with a poly-isocyanate (methylene diphenylene or toluene diisocyanate), forming the solid polymer [5,6,7].

The chemistry involved in the synthesis of rigid PUs is complex because several simultaneous reactions are involved. The simultaneity of the reactions is due to the extreme reactivity of the isocyanate group (−N=C=O) towards the hydrogen active compounds, such as those containing the −OH and −NH functional groups [6]. However, among the reactions taking place during the foaming process, two reactions are the main processes leading to the formation of a PU foam. On one hand, the *polymerization reaction* (also known as gelling) between isocyanate and polyol leads to the formation of the urethane group [1,2,3,4,5,6]. Concurrent to the gelling reaction, the isocyanate reacts with water (included in the polyol) to give an unstable carbamic acid, which spontaneously decomposes into CO_2_ (as a chemical blowing agent, CBA) and the corresponding amine in the so-called blowing reaction. Therefore, the generated gas provides the polymer expansion [1,2,3,4,5,6]. The amine may further react with another isocyanate molecule to give urea. In addition to CBAs, several physical blowing agents (PBAs) may be used as co-blowing agents to further promote the polymer expansion. Typically, PBAs are liquids with a low boiling temperature (e.g., pentane) that undergo evaporation due to the exothermicity of the reacting medium [1,2,3,4,5,6].

The polymer expansion is regulated by two bubble formation mechanisms during the early stage of the foaming process, after which bubbles grow, impinge and may eventually merge into each other, inducing coarsening on the ongoing morphology. The bubble formation mechanisms are aeration and nucleation. For aeration (also known as frothing), the gas phase (commonly air) is dispersed into the starting reactants via vigorous stirring, entrapping gas bubbles within the polymeric matrix [8]. For nucleation, according to the classical homogeneous nucleation theories, the system must overcome a thermodynamical (surface) energy barrier in order to let nucleation occur. For gas–liquid systems, once the solubility limit is exceeded, the gas dissolved into the liquid matrix precipitates in the form of metastable nuclei that may re-dissolve or grow beyond a critical radius [9]. Bubble stabilization (or, on the contrary, their collapse) may occur after the growth stage [9,10]. Aeration and nucleation may be considered competitive mechanisms: in fact, if pre-existing bubbles are already present in the reaction system (e.g., air bubbles entrapped by mechanical stirring), there is no surface energy barrier to overcome and the blowing agent evolving from CBA (or PBA) may simply inflate the pre-existing bubbles via diffusion, with no formation of additional bubbles formed via nucleation.

PU’s final properties, such as the thermal conductivity, mechanical properties, dimensional stability and fire resistance, are tightly related to the foam density and morphology. In order to tailor the final properties, it is key to study and understand the governing mechanisms in the morphology evolution, namely the balance between the gelling and blowing reaction and the physical aspects of the different stages of the process: nucleation, growing, coarsening and coalescence. In addition, the bubble size distribution and the degree of open and closed bubbles, again largely affecting the properties, should also be considered [11]. Recently, Pardo et al. used time-resolved X-ray radioscopy in order to study the bubble nucleation and growth mechanisms taking place in a small droplet (used to limit the number of cells to 2–3 maximum in the depth direction) [12]. By using this technique in combination with image analysis (IA), they were able to monitor the bubble development during the PU foaming process. In our previous work [13], we developed equipment that allowed us to monitor the different stages of the expansion process with different blowing agents. We were able to monitor the bubble size distribution as well as the bubble number density during the PU expansion. The aforementioned works clearly elucidate the progress made so far in developing equipment suitable to monitor these physical phenomena and characterize them. However, knowledge of the effects of the mixing conditions and the introduction of air bubbles into the PU reactive system on the nucleation and growth mechanisms is still poor, as well as on the reaction kinetics [14,15]. Baumhäkel et al. [10] studied the influence of the mixer stirring velocity on the final morphology of a flexible PU foam. Air bubbles were introduced in an open container by varying the mixing speed up to 3000 rpm. In this way, the authors concluded that the air bubbles introduced into the system by the mixing process accounted for all bubbles present in the final foam. A similar result was observed by Reignier et al. [15] on PU foams studied by cryogenic scanning electron microscopy. They also observed that the number of air bubbles per unit volume (~10^6^ bubbles·cm^−3^) was similar to the bubble population density of the final foam.

In the recent literature, several additives have been extensively studied and used to reduce the bubble size distribution in PU foams. For instance, solid-type nucleating agents such as talc and organoclay particles induced significant reductions in cell size, which induced complications due to the precipitation of solid particles during the foaming process and the difficulty in reaching a good dispersion [16]. Liquid-type additives such as tetramethylsilane compounds (TEMs) were also used and PU foams with a more uniform and finer cellular structure were obtained. However, these additives presented several limitations, such as high flammability, high vapor pressure and high costs [17]. In this context, this work is part of a larger project aimed at obtaining PU foams with reduced bubble sizes obtained via the introduction of novel additives. In a previous paper [18], we studied the effects of organofluorine additives (OFAs) on PU’s cellular structure, along with their relative properties. It was shown that the smaller bubble size in these foams leads to reduced thermal conductivity and slightly improved mechanical properties. These results, combined with the relative low cost of OFAs and their non-flammability [17], persuaded us to investigate the possible mechanisms induced by these compounds during the PU foaming process. To this aim, we first studied the effects of the air bubble inclusion on the nucleation and growth mechanisms during the PU foaming process in the absence of the aforementioned additives [13]. For air bubble inclusion (obtained at a high stirring velocity of 1000 rpm for 8 s), we observed that the blowing agent molecules diffuse toward these pre-existing air bubbles rather than nucleating new gas bubbles. On the contrary, in the absence of air bubbles (at a low stirring velocity of 50 rpm for 20 s), bubble nucleation was detected. Besides the investigation of these physical mechanisms, further study is needed to understand how the chemical processes are affected by the air bubble inclusion.

In the context of this project, we are interested in evaluating the effects of the air bubble inclusion on the reaction kinetics of rigid water-blown PU foams and how these affect the bubble formation and degeneration mechanisms. In the work performed by Santiago-Calvo et al. [19], the effects of functional nanofillers on the reaction kinetics of water blown PU foams were studied. To this end, the authors developed a method that allowed them to evaluate the formation of the urethane and urea groups by deconvolution of via amide I region in the infrared. In this way, it was possible to quantify the products that are representative of the gelling and blowing reactions during the foaming process. In this paper, we used this approach to follow the chemical reaction kinetics [19] combined with the bubble formation monitoring approach [13] to give a full picture of both the chemical and physical processes taking place during the foaming processes, in which different amounts of air bubbles are entrapped during the mixing stage.

## 2. Experimental

### 2.1. Materials

A formulated mixture of polyether polyols (VORACOR^TM^ CW 7028, OH number = 370, mixture density = 1.08 g/cm^3^, viscosity = 6700 mPa·s) with silicone surfactant and catalysts was utilized with polymeric methylene diphenyl diisocyanate (PMDI) (VORACOR^TM^ CE 142, 31.1% NCO, 1.20 g/cm^3^, 190 mPaּּ·s) to obtain PU foams. Both reactants were formulated and supplied by Dow Italia s.r.l. (Correggio, RE, Italy) and used “as received”. The formulated polyol and isocyanate components implemented in this study can be purchased from DOW Europe GmbH (CH). Water was always utilized as the CBA and was already contained into the polyol formulation. The composition of the formulated polyol is detailed in Table 1.

### 2.2. Optical Setup

**Figure 1:**
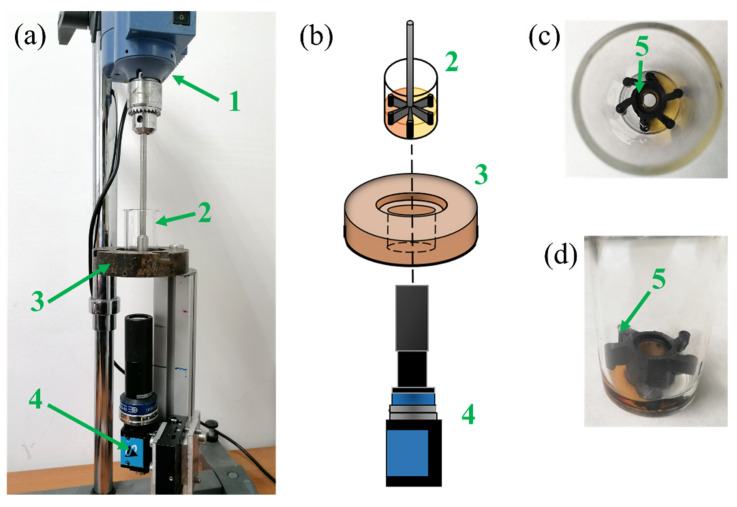
(**a**) Optical setup composed of (1) the mixing head, (2) the sample holder, (3) the copper plate used to keep the sample holder and (4) the high-speed camera. (**b**) Scheme of the optical setup. (**c**) Sample holder with (5) the rubbery impeller keeping the two reactants separate before the mixing stage and (**d**) its top view.

A simple optical observation system, already reported in [13], was used for this study. A CMOS camera (model DMK 33UX178) from The Imaging Source (Bremen, Germany) was used to collect microphotograms during the foaming process. The digital camera has a resolution of 3072 × 2048 pixel (6.3 MP) and a frame rate of 60 fps. The gain can be varied in the range of 0–48 dB and each pixel has a size of 2.4 µm × 2.4 µm. A bi-telecentric lens (model TC23004) from Opto Engineering (Mantova, Italy) was mounted (×2 magnification) with a working distance of 56 mm and a field depth of 0.23 mm. The optical setup is shown in Figure 1. In case of study of nucleation as well as aeration, the reactants were poured in a sample holder and kept separate by a rubbery impeller, as shown in Figure 1c,d. Similar amounts of polyol (1 g) and isocyanate (1 g) were mixed at several mixing speeds: 50 rpm (for 20 s), 500, 1000 and 2000 rpm (for 8 s). In this way, it was possible to monitor the mixing stage of the PU components and the foaming process in situ. The copper plate was used to keep the sample holder. The different bubble morphologies as well as the bubble size distribution were evaluated using a semi-automatic method of image analysis (IA). First of all, a region of interest (ROI) on the microphotogram was detected. Therefore, the average bubble diameter (defined as the diameter of a circle of area equivalent to the projected area of the dispersed phase) was multiplied by a correction factor of 1.273 [20] to calculate the reference three-dimensional bubble size. The bubble density was evaluated by measuring only the image area (A) and number of dispersed phases (n) according to Kumar’s theoretical approximation [21]. For each foaming experiment, the lens of the optical camera was kept on a fixed field of view. Bubbles with a defined edge with a predefined range of low and high threshold values were taken into account for the bubble density evaluation. Bubbles with edges that were not in the aforementioned range were considered out-of-field and then not included in the parameter n. Edge detection is a widely employed built-in function in many commercial IA software programs. The software utilized in this work was ImageJ [22]. In the literature, one of the most applied computational approaches for edge detection is the algorithm proposed by Canny [23] and modified by Deriche [24]. Here, the Canny edge detector was applied in order to detect the bubble candidate profiles. The IA was applied to 36 scans in the PU foaming process covering approximatively 300 s. For each formulation, the bubble size and bubble density of three samples were measured and averaged.

### 2.3. FTIR Spectroscopy

**Figure 2:**
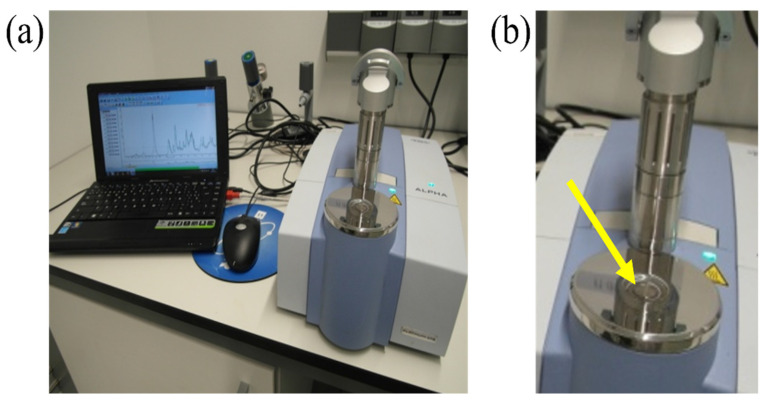
(**a**) Experimental setup used to collect the FTIR spectra and (**b**) details of the ATR cell where (see yellow arrow) the droplet of the reacting foam was poured.

A Bruker ALPHA spectrometer working in ATR mode was used to collect in situ FTIR spectra during the foaming process. The experimental setup is shown in Figure 2. As in the optical observations, similar amounts of polyol (1 g) and isocyanate (1 g) were used and the same mixing speeds were adopted. After the mixing stage, 1 mL of the reacting foam was poured on the ATR surface (shown in Figure 2b). The addition of the isocyanate was taken as time t = 0. The maximum elapsed time between the addition of isocyanate and acquisition of the first scan was approximatively 60 s. Every FTIR spectrum was taken at room temperature with a resolution of 4 cm^−1^ in the range of 4000–400 cm^−1^. Each spectrum was the average of 16 scans. Before starting the acquisition, a background spectrum was subtracted. Each acquisition lasted 120 min for a total of 240 spectra. Baseline correction was conducted in order to correct the intensity shifts at lower frequencies. After this, the asymmetric CH stretching band at 2972 cm^−1^, which according to the literature [25,26], remains constant during the process, was used as the internal reference band to account for the concentrations or density changes. The overlapped absorptions in the amide I region (carbonyl region) were deconvoluted using Gaussian bands and their relative curve fittings were adjusted using verified methods previously reported in [27,28]. The results reported herein are the averages of three experiments.

### 2.4. Foam Characterization

#### 2.4.1. Foam Density

Foam densities were measured according to the ASTM D1622/D1622M-14 standard test method [29]. The foam was cured at room temperature in the sample holder used for the optical measurements for 2 h. The foam was then removed and further cured at room temperature for at least 2 days before characterization. After this, PU samples were cut using a razor blade to measure their density. The size of the specimen was 10 mm × 10 mm × 10 mm (width × length × thickness). The densities of three samples were measured and averaged.

#### 2.4.2. Scanning Electron Microscopy (SEM)

The morphology of the final water blown PUs was studied using scanning electron microscopy (SEM) with a JSM-820 microscope (JEOL Ltd. Tokyo, Japan). The foams were cut with a razor blade and then coated with a gold monolayer under vacuum. The average bubble size (ф) and the anisotropy ratio (AR) were evaluated according to the ASTM D3576-04 standard test method [20] using IA software (ImageJ). The intersections method has been used to evaluate the ф. Lines in two perpendicular directions (m vertical lines of length h and n horizontal lines of length l) are taken in order to make a grid. The grid is overlaid in each micrograph, and for each line the number of bubbles intercepted is counted and the line length (h or l) is divided by the number of bubbles to obtain the bubble size of each line (ф_i_ or ф_j_). For each formulation, the ф and AR (calculated from Equations (1) and (2), respectively [30]) of the three samples are measured and averaged.

(1) $$ф=\frac{\sum_{i=1}^{m}{ф}_{i}+\sum_{j=1}^{n}{ф}_{j}}{m+n}$$

(2) $$\mathrm{AR}=\frac{\frac{\sum_{i=1}^{m}{ф}_{i}}{m}}{\frac{\sum_{j=1}^{n}{ф}_{j}}{n}}$$

## 3. Results and Discussion

### 3.1. Optical Acquisition

In this section, microphotograms were collected and used to evaluate the bubbles formation mechanisms, according to the procedure described in the experimental section.

#### 3.1.1. Bubble Formation Mechanisms

**Figure 3:**
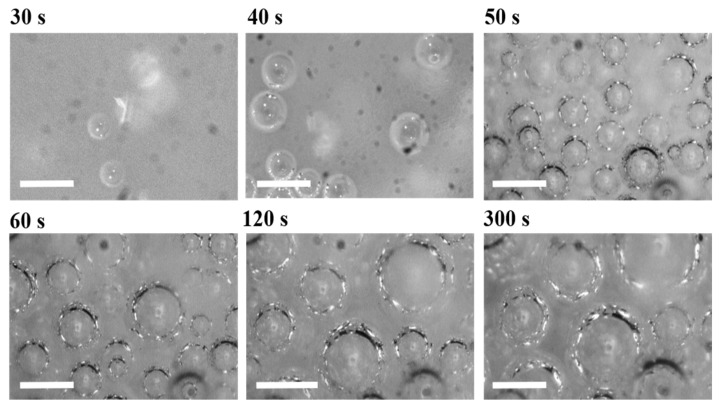
Microphotograms reporting the foaming process of the PU sample obtained at 50 rpm. Scale bars are 200 µm.

**Figure 4:**
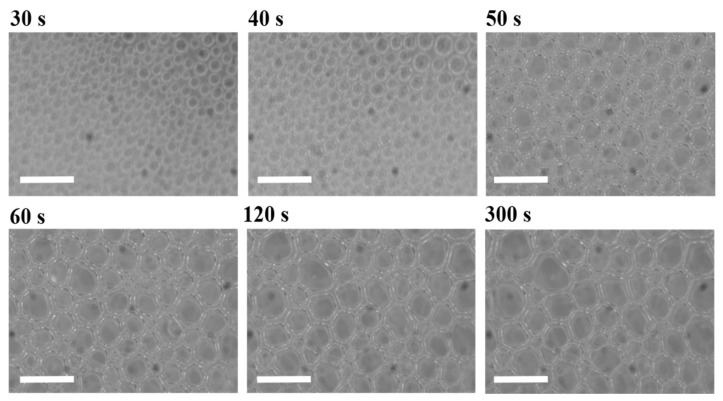
Microphotograms reporting the foaming process of the PU sample obtained at 1000 rpm. Scale bars are 200 µm.

Figure 3 and Figure 4 depict the bubble morphology evolution during the PU foaming process at 50 and 1000 rpm, respectively. From the initial microphotograms, the disparity in the amount of bubbles can be seen as a result of the different mechanisms involved in the bubble formation. The mixing conditions at 50 rpm for 20 s were found to be suitable for observing the foaming mechanisms in the absence of entrapped air bubbles. At these conditions, CO_2_ bubbles appeared to nucleate approximatively 30 s after the mixing stage and then underwent growth. On the other hand, it can be observed that the adopted mixing conditions at 1000 rpm for 8 s allowed the inclusion of many air bubbles from the very beginning of the observations (right after the mixing stage).

**Figure 5:**
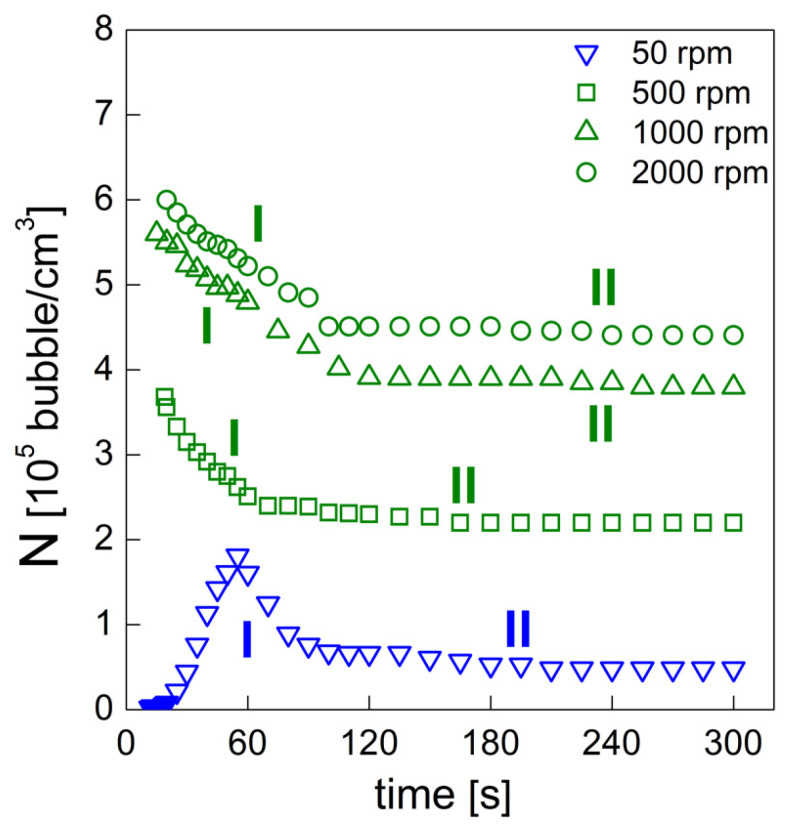
Bubble density evolution of the PU foams obtained at different mixing speeds.

The foaming behavior at the several investigated mixing speeds is reported in terms of the bubble density (N) (standard deviation ranged approximatively from 4×10^3^ bubble·cm^−3^ to 8×10^3^ bubble·cm^−3^ for each experimental point) in Figure 5. The experimental data show a significant divergence in the foaming behavior between the PU system obtained at 50 rpm and those obtained by adopting higher mixing speeds. At 50 rpm, the increase in N values during the early foaming process may be attributed to the formation of new bubbles by the BA molecules. After this, a decrease in N can be seen as the coalescence starts to occur and overcome the bubbles formation. During the foaming stage, a slighter N decrease can be observed as a consequence of further bubble degeneration. The early foaming process of the PU foams obtained at 500, 1000 and 2000 rpm is characterized by higher bubble densities as a result of the increased amount of air bubbles introduced in the mixing stage. In this case, only bubble degeneration is observed over time without any increase in N that could be attributable to the formation of new bubbles. The absence of new bubbles (possible BA nucleation) can be explained if one considers how the different nucleation mechanisms may be affected in the presence of pre-existing bubbles [29]. Bubble formation mechanisms such as homogeneous (i) and heterogeneous (ii) nucleation are processes that involve the formation of a new gas phase after overcoming a very high energy barrier (radius of the nuclei greater than the critical radius in order to let the bubble spontaneously increase) [9]. Conversely, when pre-existing bubbles are contained in the system, two additional cases may occur: when the radius of the curvature of these pre-existing bubbles is smaller than the critical radius (iii), less nucleation energy is required, while for a radius of curvature greater than the critical radius (iv), the required energy decreases to zero [31]. At mixing speeds of 500, 1000 and 2000 rpm, due to the presence of air bubbles within the reacting mixture from the very beginning (included in the mixing stage), we may infer that the BA molecules would rather diffuse toward these pre-existing air bubbles (no energy barrier to overcome, iv) than nucleate in gas bubbles into the PU matrix (i). The linear fitting of experimental data is a useful method of qualitative evaluation of the bubble formation rate (dN/dt) [32] (see Table 2). At 50 rpm, the reduction in the bubble degeneration rate during stage I can be noticed from 50 to 500 rpm (from −3095 bubble·cm^−3^·s^−1^ to −2654 bubble·cm^−3^·s^−1^ respectively), highlighting the minor degree of coalescence on the bubble coarsening. Optical observations confirmed that the bubble coarsening at 50 and 500 rpm was affected by coalescence for the overall foaming process. Interestingly, during the foaming stage I, while foams obtained at 1000 and 2000 rpm were affected by bubble coalescence (with dN/dt values being equal to −1838 bubble·cm^−3^·s^−1^ and −1601 bubble·cm^−3^·s^−1^, respectively), it was observed that during foaming stage II, the Ostwald ripening was only responsible for the bubble degeneration (with dN/dt −89 bubble·cm^−3^·s^−1^ and −69 bubble·cm^−3^·s^−1^, respectively).

#### 3.1.2. Bubble Growth

**Figure 6:**
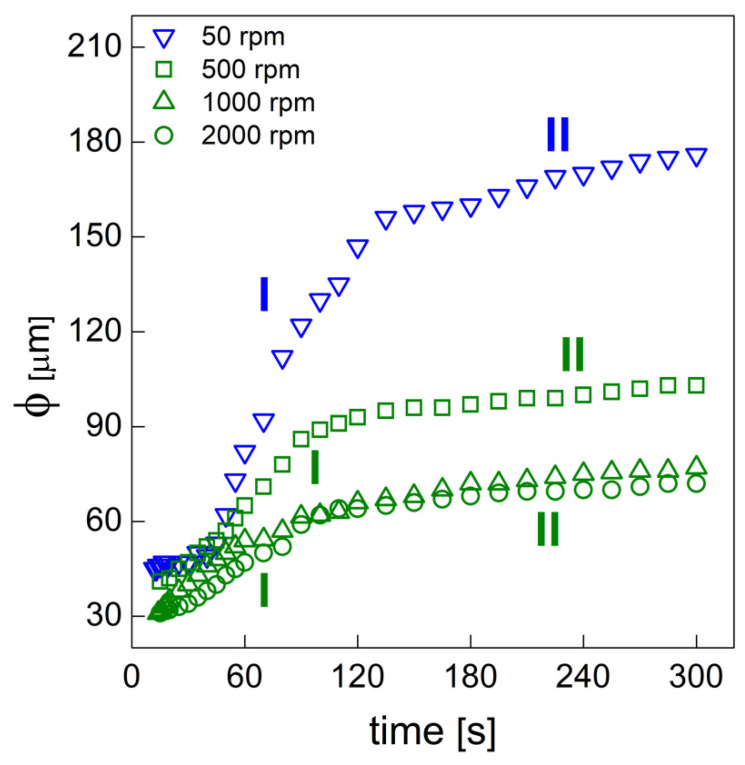
Bubble size evolution of the PU foams obtained at different mixing speeds.

After the bubble formation (nucleation or aeration) stage, gas bubbles undergo growth due to the gas diffusion from the liquid phase. Macroscopic expansion can be observed with the bubble growth. The bubble size (ф) evolution is reported in Figure 6 (the standard deviation ranged from 10 µm to 15 µm for each experimental point). For each system, we observed a two-stage bubble growth mechanism [33]. During the first stage, the experimental data showed a linear trend. During aeration, the bubble growth is caused by the inflation of the BA molecules from the polymeric matrix towards the gas bubble phase [33]. During nucleation, two mechanisms contribute to the bubble size increase: (i) the formation of new bubbles that keep appearing during the early foaming period and (ii) the growth of the previously nucleated bubbles caused by the BA inflation from the polymer phase to the gas bubble phase [33]. After stage I, rounded bubbles isolated in the liquid (wet regime) change their shape into polyhedral form and start impinging each other (dry regime), with a consequent reduction in the bubble growth rate [33]. The bubble growth rate is then reduced and a second linear trend can be observed. The data presented in Figure 6 show that foams obtained at mixing speeds of 50 and 500 rpm were characterized by a coarser cellular structure during the foaming process. The influence of the mixing speed on the polymerization rate, and thus on the reduction in the PU’s cellular structure, is described in more detail in Section 3.2.2.

As already proposed by several authors [32,34,35,36], a suitable model can be used to describe the bubble growth in expanding media. This model is developed with the assumption of assigning a specific amount of liquid to each individual bubble in a liquid medium containing a large number of gas bubbles. A single bubble is considered spherical and surrounded by a concentric liquid shell. The gas bubble can be composed of a pure compound or a mixture of several components, while the liquid in the envelope is a solution that contains the same components at a uniform initial concentration [35]. Based on this assumption, we may define an apparent diffusivity Ɗ_a_ by taking into account the relative diffusion of these components and extrapolating this term from Equation (3) [32,33,36]:

(3) $$ф=6{\left(\frac{3}{4\pi }\right)}^{0.33}\;{\left({Ɗ}_{a}t\right)}^{0.5}\;$$

A reduction in Ɗ_a_ (the results are reported in Table 3) can be noticed for each mixing speed when the cellular structure evolves from stage I to stage II through the impingement. During stage I, polymeric chains of the thermosetting material are characterized by a higher degree of mobility due to a lower molecular weight, while after the impingement this mobility is hindered and restricted due to the ongoing curing process. Therefore, for each foaming system, Ɗ_a_ is further decreased during stage II [33].

**Figure 7:**
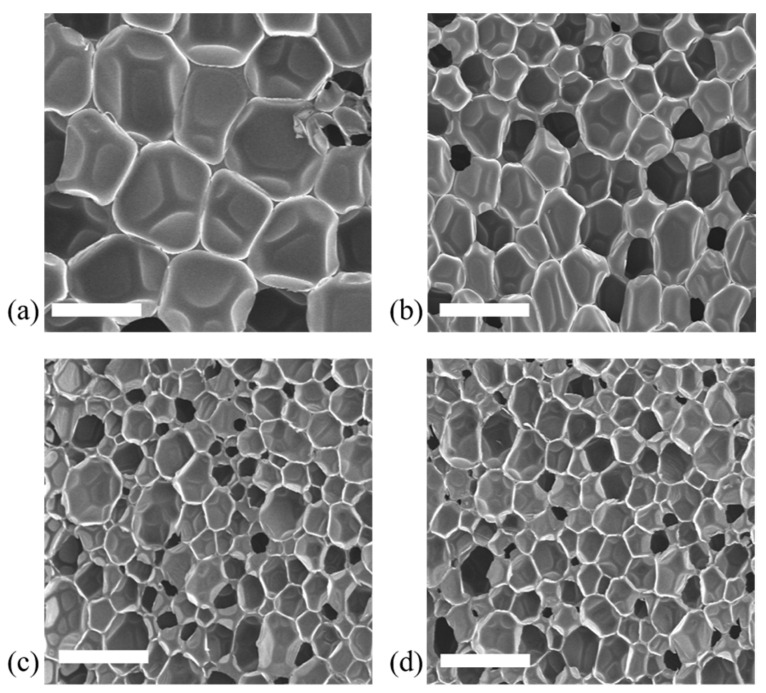
SEM micrographs of PU samples obtained at (**a**) 50, (**b**) 500, (**c**) 1000 and (**d**) 2000 rpm. Scale bars are 200 µm.

The morphology of the PU foams obtained at different mixing speeds is shown in the SEM images reported in Figure 7. From the SEM micrographs, it was possible to measure the average bubble size (ABS) and anisotropy ratio (AR) and to evaluate the bubble size distribution (see Table 4). For the different formulations under study, it is evident that the average bubble size is reduced and is more uniform (lower standard deviation) when the mixing speed is increased. Moreover, it can be seen that the density decreases with increasing mixing speed. These results can be explained by taking into account the different actions of the mixing conditions on the reactivity of the PU mixture. We may speculate that at low mixing speeds, the polymerization process responsible for the viscosity increase in the polymer melt [6] cannot compete with the blowing reaction responsible for the polymer expansion [6], resulting in coalescence affecting the overall PU foaming. With increased mixing speed, the reactants undergo more suitable mixing, resulting in a balanced process. Moreover, the variation in AR can be considered negligible, indicating that the mixing speeds do not affect the cellular structure or orientation.

### 3.2. FTIR Spectroscopy

In this section, FTIR spectra are used to monitor the concurrent gelling and blowing reactions. The isocyanate, which is readily detectable by FTIR, is involved in both reactions and may be used to evaluate the overall reaction rate by following its consumption [19]. The products of these reactions, namely the urethane and urea, are also readily detectable by FTIR and their production rates may be quantified by deconvolution analysis of the amide I region [19]. Spectra shown herein were normalized according to the procedure described in the experimental section.

#### 3.2.1. Isocyanate Conversion

**Figure 8:**
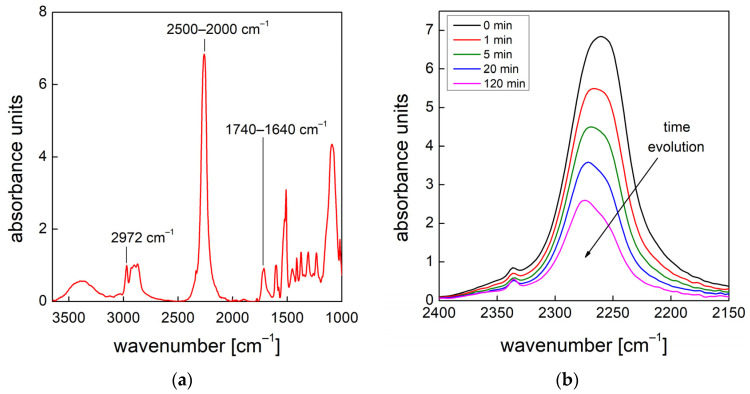
(**a**) FTIR spectrum of the PU reaction mixture obtained at 1000 rpm collected right after the mixing step. (**b**) Details of the monitored peaks of the NCO characteristic group (2500–2000 cm^−1^).

Figure 8a reports the FTIR spectrum of the PU reaction mixture at 1000 rpm right after the mixing step of the PU reactants. We also report details (characteristic peak wavenumbers) of the spectral regions of interest used to evaluate the consumption and production rates. The isocyanate conversion can be evaluated by monitoring the band of the characteristic NCO group in the range of 2500–2000 cm^−1^ [19] (Figure 8b).

**Figure 9:**
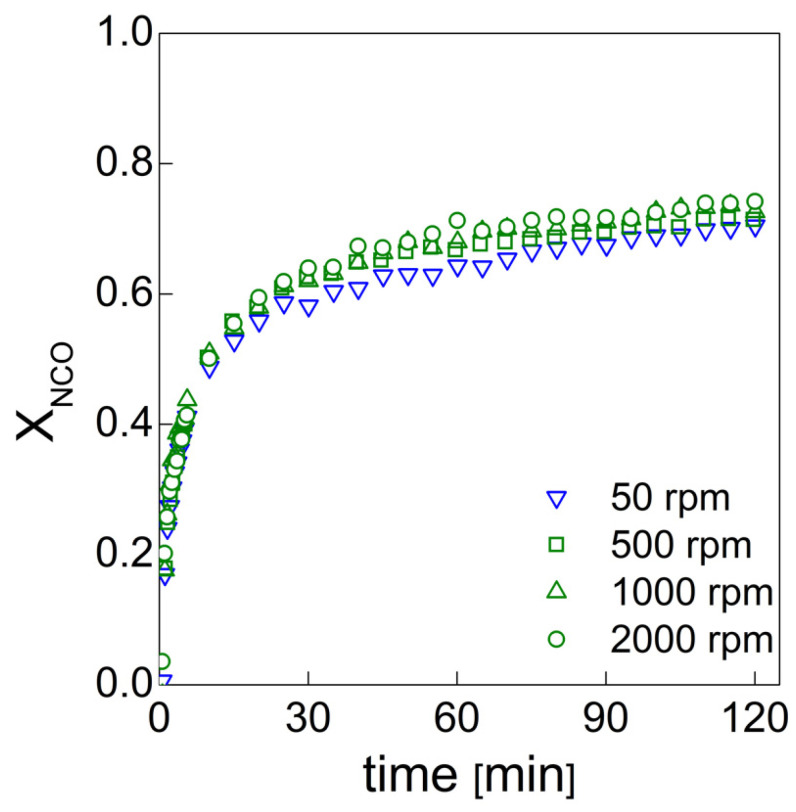
Isocyanate conversion as a function of time for the systems obtained at the different mixing speeds.

The absorbance area under the NCO peak can be integrated and the following equation can be used to obtain the isocyanate conversion [26,37]:

(4) $$X_{\mathrm{NCO}}=\frac{A_{\mathrm{NCO}}^{0}-A_{\mathrm{NCO}}}{A_{\mathrm{NCO}}^{0}}$$

where $A_{\mathrm{NCO}}^{0}$ and A_NCO_ are the integrated absorbance areas under the NCO peak at times 0 and t (current time of acquisition), respectively. Figure 9 shows the isocyanate conversion for the systems under study at 50, 500, 1000 and 2000 rpm. For each experiment, the isocyanate is not completely consumed, even after 2 h. Moreover, from the comparison a slightly mixing speed effect can be noticed on the final isocyanate conversion.

Typically, the reaction kinetics of thermosetting polymers is described by various kinetic models. In this case, the reaction rate can be described by a phenomenological model frequently used for homogeneous and heterogenous processes that are governed by phase boundary reaction mechanisms, yielding an assumption of a first-order model [37,38]:

(5) $$X_{\mathrm{NCO}}=X_{\mathrm{NCO}}^{f}\;\left(1-e^{-\mathrm{kt}}\right)$$

where X_NCO_ and $X_{\mathrm{NCO}}^{f}$ are the time-dependent relative conversion and its final value, respectively, while k is the Arrhenius-type kinetic constant ($k={\mathrm{Ae}}^{-E_{a}/\mathrm{RT}}$, A, E_a_, R, T being the pre-exponential factor, activation energy, ideal gas constant and temperature, respectively). In our case, the one-exponential model led to significant deviation of the fitting from the experimental data. The two-exponential model was proven to be more accurate in the fitting of these data (Equation (6)). This better agreement with experimental data was due to the onset of the gel point. Before the gel point, the reaction rate is chemically controlled as the polymeric chains are characterized by a higher degree of mobility due to the lower molecular weight. After the gel point, this mobility is hindered and restricted, and the process becomes diffusion-controlled [39]:

(6) $$X_{\mathrm{NCO}}=X_{\mathrm{NCO}}^{f}\;\left(1-{a\;e}^{-k_{1}t}-{b\;e}^{-k_{2}t}\right)$$

where a and b are parameters used to fit the experimental data, k_1_ and k_2_ are the Arrhenius-type kinetic constants representing the chemically controlled and diffusion-controlled periods, respectively. The results (i.e., final conversion $X_{\mathrm{NCO}}^{f}$, the kinetic constants k_1_ and k_2_, their relative ratio k_1_/k_2_ and the fitting parameters a and b together with R^2^) of the fitting procedure are collectively reported in Table 5. The kinetic constants, k_1_ and k_2_, have several values that seem to not exhibit an evincible trend with the mixing conditions; moreover, the k_1_/k_2_ ratio maintains the same order of magnitude. On the other hand, the final conversion, $X_{\mathrm{NCO}}^{f}$, slightly increases with the mixing speed, as more isocyanate is consumed. From the analysis of these values, we can conclude that the mixing conditions improve the isocyanate consumption, while they do not seem to indicate effects on the ongoing gel point of the PU reaction mixture. Finally, a and b are modulation factors used to optimize the double exponential function and to maintain similar values for each fitting procedure.

#### 3.2.2. Deconvolution of the Amide I Region

**Figure 10:**
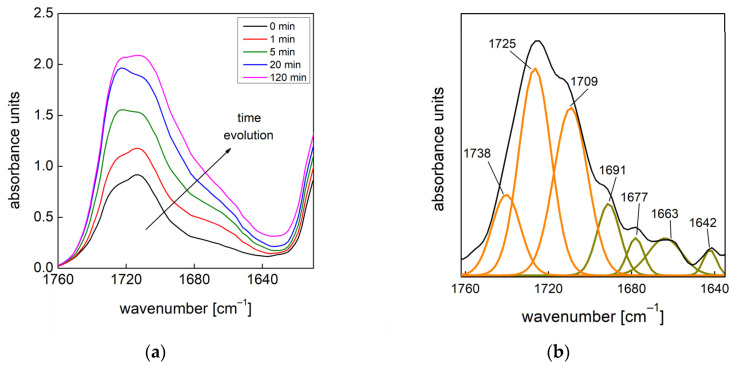
(**a**) Details of the monitored peaks of the amide I region (1740–1640 cm^−1^) of PU foam obtained at 1000 rpm. (**b**) Example of deconvolution of the amide I region after 20 min via curve-fitting procedure.

The urethane and urea formation can be evaluated by deconvolution of the amide I region in the range of 1740–1640 cm^−1^ [19]. The frequencies assigned to the typical vibrations of the different C=O carbonyl groups are shown in Figure 10b (FTIR spectrum of PU obtained at 1000 rpm) and reported in Table 6. As shown, seven peaks can be taken for the analysis: three (orange) peaks on the left side of the region are used for the quantitative evaluation of the urethane and four (dark yellow) peaks on the right side of the region are used for the quantitative evaluation of the urea.

**Figure 11:**
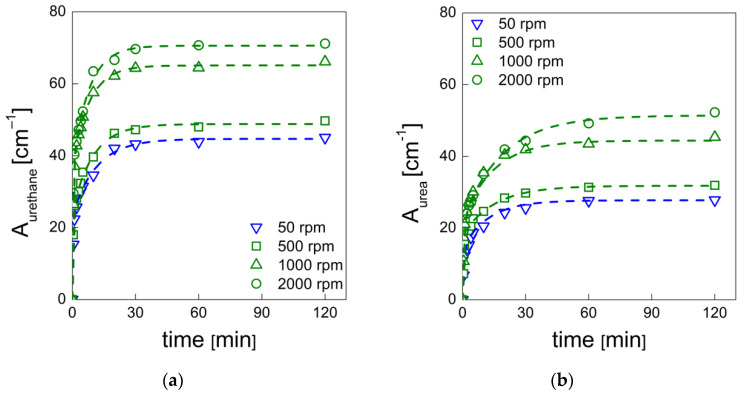
(**a**) Urethane and (**b**) urea formation obtained by deconvolution procedure as a function of time. Dots represent experimental data, dashed lines represent fitting of the experimental data by Equation (7).

At each time, the total urethane formation can be calculated via the sum of the integrated absorbance areas related to all urethane peaks ($A_{\mathrm{urethane}}=\sum A_{C=O}^{\mathrm{urethane}}$). Likewise, the total urea formation can be calculated by the sum of the integrated absorbance areas related to all urea peaks ($A_{\mathrm{urea}}=\sum A_{C=O}^{\mathrm{urea}}$). As the isocyanate consumption is involved in more concurrent reactions, it is easier to evaluate the effects of the mixing speed on the reaction kinetics in terms of urethane and urea formation. The assumptions adopted to derive the kinetic model of Equation (6) in this case yield [37,38,39]:

(7) $$A_{\mathrm{product}}=A_{\mathrm{product}}^{f}\;\left(1-{a\;e}^{-k_{1}t}-{b\;e}^{-k_{2}t}\right)$$

where A_product_ is the integrated absorbance area of the total urethane (A_urethane_) formation or the total urea formation (A_urea_) at time t, and $A_{\mathrm{product}}^{f}$ is the final value of integrated absorbance area. Data obtained by deconvolution analysis and fitting by Equation (7) for selected mixing speeds are reported in Figure 11a,b, while the results of the fitting procedure are reported in Table 7 and Table 8.

$A_{\mathrm{Urethane}}^{f}$ and $A_{\mathrm{Urea}}^{f}$ increase with the mixing speed in accordance with the increase already observed for $X_{\mathrm{NCO}}^{f}$. In fact, the increase in isocyanate conversion (reactant) corresponds to increases in the urethane and urea formation (products). Typically, the reactant molecules have to hit each other in an efficient way in order to let the reaction occur [40]. In our case, with higher mixing speeds, a more suitable contact of the reactants is allowed, and as a consequence there is an enhanced probability of collisions of these molecules. In this way, boosted reaction kinetics may be observed for both polymerization and blowing [40]. It is noteworthy that besides the polymerization and the blowing reactions (the main processes that characterize the PU foaming process), the isocyanate can react with other compounds to allow the formation of other products as well. Among these, the isocyanate may react with additional-NH functional groups to give allophanate as well as biuret compounds, and may be further involved in dimerization and cyclotrimerization reactions to give dimers and trimers [6]. As a consequence, while urethane and urea formation show almost no changes during the foaming process (Figure 11a,b), isocyanate conversion exhibits a slow increase due to the aforementioned chemical reactions (Figure 9).

The kinetics constants k_1_ and k_2_ maintain the same order of magnitude so that the k_1_/k_2_ ratio does not undergo a large variation. Regarding product formation, it can be observed that the diffusion-controlled kinetics seems to exert a major influence on the ongoing gel point with the increasing mixing speed. This behavior can be evinced by k_1_/k_2_ values, which in case of urea formation keep decreasing, while this occurrence may not be extrapolated from data for urethane formation. Finally, a and b are also modulation factors used to optimize the double exponential function and maintain similar values for each fitting procedure.

Data retrieved from the analysis conducted on the reaction kinetics of PU foaming, at the different mixing speeds, can be used to correlate the observed bubble degeneration with the urethane and the urea formation, which in turn provide indirect measures of the polymerization and the blowing reaction rates, respectively [6]. During the early foaming stage (stage I), it was observed that coalescence less affected the bubble collapse with the increasing mixing speed. In fact, dN/dt decreased from −3095 to −1601 bubble.cm^−3^.s^−1^ when evaluating bubble degeneration rates ranging from 50 to 2000 rpm. respectively. Here, we can infer that at low mixing speeds, the gelling reaction is inhibited by the indigent mixing conditions, meaning the polymer cannot withstand the bubble expansion and relative coarsening. To further elucidate this observation, we performed a more appropriate analysis by expressing the degree of urethane (and urea) formation as a normalization process of the product-integrated absorbance area with respect to its final value, as follows [38]:

(8) $$X_{\mathrm{urethane}}=\frac{A_{\mathrm{urethane}}}{A_{\mathrm{urethane}}^{f}}=\left(1-{a\;e}^{-k_{1}t}-{b\;e}^{-k_{2}t}\right)$$

(9) $$X_{\mathrm{urea}}=\frac{A_{\mathrm{urea}}}{A_{\mathrm{urea}}^{f}}=\left(1-{a\;e}^{-k_{1}t}-{b\;e}^{-k_{2}t}\right)$$

**Figure 12:**
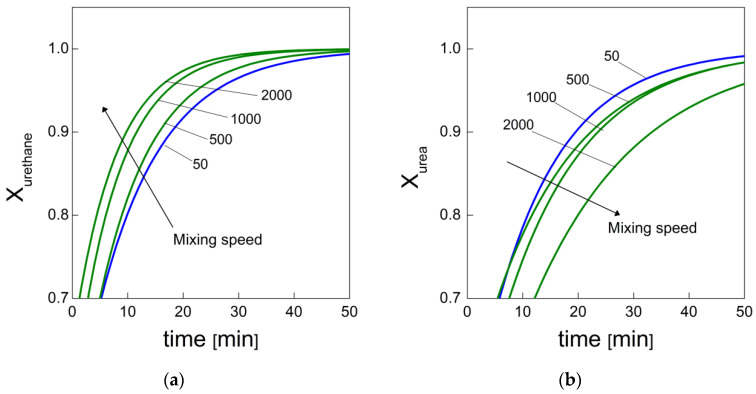
(**a**) Degree of urethane formation and (**b**) degree of urea formation reacted at different mixing speeds as a function of reaction time.

Figure 12 reports the normalized curves obtained by Equations (8) and (9). Indeed, one can notice that the degree of urethane and the degree of urea always reach their final values (completion of the reactions). However, on one hand, the time taken for the urethane formation rate to reach the completion of reaction decreases with the mixing speed, while on the other hand the time taken for the urea formation rate increases.

The opposite trends between the two product formation rates strengthen the observation that the higher the mixing speed, the faster the polymerization rate so that the coalescence is progressively depleted [13,14,15]. Moreover, the trade-off between the polymerization and the blowing reactions, in favor of the former, influences the attainment of the gel point as well. From results obtained via fitting procedures implemented with Equation (7), we can observe that k_1_/k_2_ goes from 0.0567 to 0.0326 (from 50 to 2000 rpm, respectively) for urea formation, indicating that a shorter time elapsed for the PU reaction to attain the gel point, which indicates the transition from a chemically controlled reaction to a diffusion-controlled one [39]. In addition, the observed trend is also in good agreement with the identified time intervals in which dN/dt and Ɗ_a_ were evaluated. Accordingly, the transition between stages I and II can be associated with the attainment of the gel point [39]. The increasing viscosity during the PU thermosetting reaction leads to reduced molecular diffusion of the species contained in the reaction system that terminate after the gel point [41]. The molecular diffusion associated with inter-molecular movements can be adequately described by data related to the apparent diffusivity, Ɗ_a_ [32,33,39,41]. In fact, for stage I, the results obtained from Equation 3 show that Ɗ_a_ goes from 2.95×10^−5^ to 2.22×10^−6^ cm^2^/s when going from 50 to 2000 rpm, respectively. However, the reaction continues even after the gel point through intra-molecular interactions that are associated with local movements of the polymeric branches [41]. In stage II, Ɗ_a_ goes from 9.92×10^−7^ to 1.09×10^−7^ cm^2^/s when going from 50 to 2000 rpm, respectively. Indeed, the combined results for the apparent diffusivity as well as the gel point effectively confirm that the molecular diffusion is reduced when higher mixing speeds are adopted, leading to more contained bubbles growth during the foaming process [32,33]. Moreover, it is also highlighted that the coalescence is reduced when the polymer matrix is characterized by a higher polymerization rate in contrast to a lower blowing reaction rate [13,14,15], as also confirmed by FTIR results. We observe that for foams obtained at 1000 and 2000 rpm, the Ostwald ripening appears to be responsible for the further cell coarsening in the late foaming process. The design of experiments to elucidate which mechanisms regulate the occurrence of the Ostwald ripening in the PU foaming will be subject of a forthcoming paper.

## 4. Conclusions

PU bubble formation mechanisms and reaction kinetics at several mixing speeds were studied using in situ micro-optical observations and FTIR spectroscopy, respectively. The bubble size, evolution and density were determined via image analysis, while gelling and blowing reactions were quantified by deconvolution analysis of the amide I region. At 50 rpm, right after the mixing stage, no air bubbles are included in the PU reaction system. New CO_2_ bubbles appear to nucleate during the early foaming process. In this case, as with the product formation rate given via FTIR data, the gelling reaction is inhibited by the indigent mixing conditions, meaning the polymer cannot withstand the bubble expansion and the relative coarsening. Indeed, after the nucleation stage, these bubbles undergo coalescence and this degeneration mechanism affects the overall foaming process. At 500 rpm, air bubbles are included in the PU reaction system and no formation of new bubbles was observed during the foaming process. After the mixing stage, these bubbles undergo growing caused by CO_2_ diffusion. For this PU system, the gelling reaction is not strongly depleted, as in the case at 50 rpm, but still the polymer is not capable of withstanding the bubble expansion, meaning the coalescence still affects the overall foaming process. At 1000 and 2000 rpm, bubble degeneration was affected by coalescence during the early foaming stage and by Ostwald Ripening in the late foaming process. In any case, PU samples obtained at 1000 and 2000 rpm were characterized by a finer bubble size distribution when compared to samples obtained at 500 rpm, and were also affected by coalescence. For these samples, the polymerization was enhanced by the higher stirring velocity, which allowed us to obtain more suitable mixing conditions. In this way, the PU foaming results in a balanced process between the bubbles inflation and the viscosity increase.

In summary, these observations reflect how the different mixing conditions can affect the overall reaction kinetics. The final cellular structure of the product can also be modulated as a consequence of the different selected mixing speeds. Based on this approach, by coupling optical observation and FTIR spectroscopy processes, one can adequately choose the process conditions in order to obtain the desired PU foam that is suitable for the appropriate industrial application.

## Figures and Tables

**Table 1:** Chemical composition of the formulated polyol.

Chemicals	Description	Parts
Polyol	Mixture of polyether polyols	95.4
Surfactant	Silicone type	2.8
Catalysts	Amine type	1.8
CBA	Water	0.1

**Table 2:** Results of the fitting procedure.

Mixing Speed [rpm]	Stage I		Stage II	
	dN/dt [Bubble·cm^−3^·s^−1^]	R^2^	dN/dt [Bubble·cm^−3^·s^−1^]	R^2^
50	−3095 (60–90 s) *	0.966	−110 (90–300 s)	0.982
500	−2654 (10–60 s)	0.957	−90 (60–300 s)	0.952
1000	−1838 (10–60 s)	0.991	−89 (100–300 s)	0.939
2000	−1601 (10–60 s)	0.989	−69 (100–300 s)	0.935

* Time intervals in which the linear fitting was applied.

**Table 3:** Results of the fitting procedure caused by Equation (3).

Mixing Speed [rpm]	Stage I		Stage II		Coarsening in Stage I/Stage II
	Ɗ_a_ [cm^2^/s]	R^2^	Ɗ_a_ [cm^2^/s]	R^2^	
50	2.95 × 10^−5^ (60–120 s) *	0.962	9.92 × 10^−7^ (120–300 s)	0.983	Coalescence/Coalescence
500	6.18 × 10^−6^ (10–60 s)	0.987	1.52 × 10^−7^ (60–300 s)	0.947	Coalescence/Coalescence
1000	2.25 × 10^−6^ (10–60 s)	0.956	2.73 × 10^−7^ (100–300 s)	0.982	Coalescence/Ostwald Ripening
2000	2.22 × 10^−6^ (10–60 s)	0.966	1.09 × 10^−7^ (100–300 s)	0.948	Coalescence/Ostwald Ripening

* Time intervals in which the linear fitting is applied.

**Table 4:** Features of the PU samples used for the optical acquisition.

Mixing Speed [rpm]	ABS [µm]	AR	Density [kg/m^3^]
50	173 ± 10	0.96 ± 0.05	58.3 ± 8.4
500	94 ± 7	0.99 ± 0.07	51.6 ± 1.2
1000	63 ± 6	0.97 ± 0.08	43.2 ± 0.9
2000	62 ± 3	0.97 ± 0.07	42.4 ± 0.9

**Table 5:** Effects of mixing speed on NCO conversion: results of the fitting procedure.

Mixing Speed (rpm)	$X_{NCO}^{f}$	K_1_ × 10^−2^ [min^−1^]	K_2_ × 10^−3^ [min^−1^]	K_1_/K_2_	a	b	R^2^
50	0.689	0.792	0.520	15.2	0.653	0.421	0.990
500	0.714	0.899	0.773	11.6	0.642	0.465	0.992
1000	0.728	0.488	0.336	14.5	0.679	0.323	0.990
2000	0.756	0.566	0.376	15.1	0.641	0.344	0.993

**Table 6:** Frequencies and band assignments of the carbonyl groups in the amide I region.

Functional Group	Frequency (cm^−1^)	Band Assignment
(C=O) Urethane	1740–1730	Free urethane [19,32,33,34]
	1730–1725	Hydrogen-bonded urethane HS */SS ** interaction [32,33,34]
	1715–1700	H-bonded urethane HS/HS interaction [32,33,34]
(C=O) Urea	1700–1690	Free Urea [18,19,32,35]
	1690–1650	Disordered hydrogen-bonded urea [32]
	1670–1660	Hydrogen-bonded C=O and N-H to an ether group [35]
	1650–1640	Ordered hydrogen-bonded urea [18,19,32,35,36]

* HS = Hard Segment; ** SS = Soft Segment.

**Table 7:** Effect of mixing speed on urethane formation: results of the fitting procedure.

Mixing Speed (rpm)	$A_{Urethane}^{f}$ (cm^−1^)	k_1_ [min^−1^]	k_2_ [min^−1^]	k_1_/k_2_	a	b	R^2^
50	44.7	0.087	1.929	0.0451	0.472	0.528	0.996
500	48.8	0.103	2.541	0.0405	0.501	0.500	0.995
1000	65.1	0.125	2.227	0.0561	0.426	0.574	0.998
2000	70.6	0.130	2.939	0.0442	0.482	0.519	0.996

**Table 8:** Effect of mixing speed on Urea formation: results of the fitting procedure.

Mixing Speed (rpm)	$A_{Urea}^{f}$ (cm^−1^)	k_1_ [min^−1^]	k_2_ [min^−1^]	k_1_/k_2_	a	b	R^2^
50	27.7	0.0802	1.415	0.0567	0.478	0.530	0.992
500	31.8	0.0653	1.309	0.0499	0.428	0.586	0.989
1000	44.4	0.0687	1.425	0.0482	0.504	0.499	0.994
2000	51.4	0.0519	1.593	0.0326	0.563	0.440	0.996

## Data Availability

The data that support the findings of this study are available from the corresponding author upon reasonable request.
